# Communication practices and challenges of healthcare providers in febrile seizure counseling: A cross-sectional study

**DOI:** 10.1097/MD.0000000000051284

**Published:** 2026-10-23

**Authors:** Laila A. Albishi, Aseel Hameed Algohani, Reem Fayez J. Alanazi, Layan Mohammed M. Alamri, Ftoon Sattam A. Albalawi, Raghad Ibrahim H. AlOmari, Ammar Mohammed Aeid, Rana Nafea Alanazi, Meshari Abdulrahman Y. Alharthi, Bdour Awad Almasoudi

**Affiliations:** a Pediatric Department, Faculty of Medicine, University of Tabuk, Tabuk, Saudi Arabia.

**Keywords:** communication, counseling, febrile seizures, healthcare providers, parent education

## Abstract

Febrile seizures (FS) frighten parents, but few studies have examined how healthcare providers counsel families. We described providers’ FS communication practices, confidence, challenges, and resource needs, and how these varied with provider characteristics. In this cross-sectional survey, 150 healthcare providers (81 pediatricians, 46 nurses, 14 general practitioners, and 9 emergency physicians) at 5 facilities in Tabuk, Saudi Arabia, completed a questionnaire. Descriptive statistics used 95% confidence intervals (CIs), chi-square and Kruskal–Wallis tests, and Firth, logistic, and ordinal logistic regression. Most providers used communication guidelines (85.3%) and verbal explanation (92.0%); other teaching methods were each used by <12.0%. After adjustment, >10 years of experience was associated with guideline use (adjusted odds ratio [aOR] 6.97, 95% CI 2.08–30.08) and higher confidence (aOR 4.20, 95% CI 2.08–8.52), and >5 FS cases/month with guideline use (aOR 4.04, 95% CI 1.54–11.46). Pediatricians reported higher confidence than other professions (aOR 2.40, 95% CI 1.27–4.54). Providers in pediatric hospitals more often had formal communication training (aOR 2.44, 95% CI 1.17–5.08), and those in private clinics/hospitals counseled longer (aOR 3.81, 95% CI 1.35–10.79). The most frequent challenges were misinformation (43.3%), cultural beliefs (42.7%), and strong parental emotions (39.3%). Providers most often asked for better educational materials (53.3%) and standardized guidelines (52.0%). FS counseling relied mainly on verbal explanation. Experience and FS exposure were associated with guideline use, profession and experience with confidence, and workplace with training and counseling time. Whether standardized guidelines and better materials improve parents’ understanding or anxiety needs testing in studies of parent outcomes.

## 1. Introduction

Febrile seizures (FS) are the most common type of convulsion in the pediatric population, affecting children between 6 months and 5 years of age in the absence of underlying neurological disease.^[1]^ While FS are benign and self-limited conditions, they often trigger intense fear and psychological distress for parents, leading to an emotional and practical burden on families.^[2,3]^ Data showed that parental anxiety, misconceptions, and inappropriate 1st-aid responses influenced clinical decision-making and healthcare utilization, supporting the need for focused educational interventions.^[4]^ Parental concerns extended beyond acute events to include worries about long-term neurological outcomes, recurrence risk, family functioning, and the child’s well-being, underscoring the public health implications of inadequate caregiver understanding.

A critical component of effective FS management is parental education, as evidence demonstrates that improving caregiver knowledge reduces anxiety, enhances coping, and encourages the use of appropriate healthcare resources.^[5,6]^ Conversely, insufficient knowledge about FS causes, characteristics, and expected course has been strongly associated with more parental distress and maladaptive responses.^[7]^ Therefore, closing these gaps between healthcare providers and caregivers through effective communication is essential. Studies have shown that counseling parents with structured information, reassurance, and emotional support improves parental confidence, reduces fear, and promotes appropriate management during recurrent FS.^[8]^ A clear explanation of disease features, particularly the estimated recurrence risk of 30% to 50%, is an essential element of counseling.^[9]^

A recent systematic analysis examining the nonclinical burden of FS^[10]^ highlighted persistent gaps in parental knowledge and emphasized the value of targeted educational strategies. The review demonstrated that healthcare-provider-led interventions can meaningfully reduce anxiety, correct misconceptions, and improve 1st-aid practices. The authors concluded that comprehensive parental education should include FS characteristics, expectations for recurrence, and practical steps for responding to future events.

Despite growing recognition of parents’ educational needs, most existing studies have focused primarily on caregiver knowledge, parental anxiety, and interventions for parents, rather than on healthcare providers’ communication behaviors when delivering this education. Very few studies have examined how providers structure their counseling, which communication methods they use, how confident they feel when educating families, or the barriers they face during these discussions.^[11]^ Moreover, there is little evidence describing the specific strategies providers use to address parental anxiety, despite its central role in determining parental understanding and management of FS. A small number of studies have assessed providers’ clinical knowledge or adherence to FS guidelines, but none address communication quality or provider-reported needs.^[12]^

To address this gap, the present study examines healthcare providers’ communication practices, confidence levels, perceived challenges, and resource needs when counseling parents about febrile seizures. It also examines how these vary with profession, clinical experience, workplace, and febrile seizure exposure, to identify where training and educational resources may be most needed.

## 2. Methods

### 2.1. Study design, setting, and population

This cross-sectional survey evaluated communication practices, confidence levels, perceived challenges, and resource needs among healthcare providers who counsel parents about febrile seizures. The study was conducted from April to August 2025 at 5 hospitals and primary healthcare centers in Tabuk city, chosen to include general hospitals, pediatric hospitals, and private clinics/hospitals.

Eligible participants included pediatricians, general practitioners, emergency physicians, and nurses directly involved in the diagnosis, management, or parental counseling of children presenting with FS. Within these facilities, providers were recruited by convenience sampling. Providers not engaged in pediatric clinical care or who lacked parent interaction responsibilities were excluded.

A minimum sample size of 135 was required to detect medium effect sizes (Cohen *w* = 0.3) for chi-square tests with 4 predictor variables, at 80% power and a 5% significance level. To account for possible incomplete responses, the target sample was increased by 10%. A total of 150 participants completed the survey, exceeding the required minimum.

### 2.2. Questionnaire development, validity, and scoring

No validated instrument measures how healthcare providers counsel parents about febrile seizures, so we developed a study-specific questionnaire. We based the items on published work on parental febrile seizure education,^[5,6,10]^ surveys of providers’ febrile seizure knowledge and practice,^[11,12]^ and a communication skills framework.^[13]^ Three pediatric specialists and 2 medical educators reviewed the draft for relevance, clarity, and coverage of the counseling process. We then piloted it with 15 healthcare providers, who were not included in the final sample, to check the clarity and relevance of the wording and response options.

The final questionnaire had 14 items in 4 sections (Supplemental Digital Content 1, Supplemental Digital Content 1). The 1st section covered demographic data: profession, years of clinical experience, primary workplace, and FS seen per month. The 2nd covered knowledge and confidence: confidence in educating parents, guideline use, key points covered, and formal communication training. The 3rd covered communication strategies: teaching methods, counseling time, how providers check parents’ understanding, and the feelings they most often encounter in these discussions. The 4th asked about the challenges providers face and the resources they need.

Confidence in educating parents was a single item rated from 1 (not confident) to 5 (very confident) and analyzed as an ordinal variable. Guideline use was a yes/no item. Counseling time had 4 ordered categories: <5, 5 to 10, 10 to 15, and >15 minutes. Formal communication training had 4 categories: no training at all, no formal training (experience-based), training during medical or nursing school, and workshops or continuing medical education. For regression, the last 2 were grouped as any formal training and the 1st 2 as none. Key points covered, teaching methods, challenges, and resource needs were check-all-that-apply items. Each option was coded 0 or 1 and is reported as the percentage of all 150 respondents who selected it, so these percentages add to more than 100%. All other items allowed 1 answer. Because confidence was a single item, and the challenge and resource questions are checklists of separate options rather than multi-item scales, we did not calculate internal consistency coefficients.

### 2.3. Data collection procedure and outcome measure

Data were collected anonymously using an online form. The questionnaire link was shared with healthcare providers at the participating facilities by department heads and through departmental WhatsApp groups. Because the link could be forwarded, the number of providers who received it, and therefore the response rate, could not be determined. Participation was voluntary, and completing the questionnaire was taken as informed consent. No identifiable information was recorded. The outcome measures included primary outcomes such as confidence in educating parents, use of clinical communication guidelines, time dedicated to parent education, and receipt of formal communication training. Secondary outcomes included provider-reported communication challenges and desired resources to enhance FS counseling.

### 2.4. Statistical analysis

Categorical variables are summarized as frequencies and percentages with Wilson 95% confidence intervals (CIs). We 1st tested associations between 4 provider characteristics (profession, experience, workplace, and febrile seizure cases per month) and 4 outcomes (guideline use, formal training, confidence, and counseling time) without adjustment. Because some expected cell counts were below 5, we compared guideline use and formal training (4 categories) with Monte Carlo exact chi-square tests (20,000 permutations). Confidence and counseling time were compared with Kruskal–Wallis tests and, when these were significant, with Dunn pairwise tests and Bonferroni correction.

To account for potential confounding between experience, workplace, and FS exposure, we then fitted 1 multivariable model for each outcome, entering all 4 provider characteristics together. We combined categories so that the number of parameters suited the number of outcome events: profession (pediatrician vs other), experience (<5, 6–10, and >10 years), workplace (general hospital, pediatric hospital, and private clinic/hospital), and FS cases (<5 vs >5/mo). Only 22 providers did not use guidelines, and every provider seeing 11 or more cases per month used them, so we modeled guideline use with Firth penalized likelihood logistic regression and profile likelihood CIs. We used binary logistic regression for any formal training and ordinal logistic regression for confidence and counseling time (4 categories). For confidence, scores 1 and 2 were combined because only 6 providers chose 1. We checked the proportional odds assumption with the Brant test and multicollinearity with variance inflation factors. In sensitivity analyses, we refitted guideline use with standard logistic regression, confidence with a 5-level ordinal model, and confidence (4 or 5 vs lower) and counseling time (10 minutes or more vs less) with binary logistic regression. We report adjusted odds ratios (aOR) with 95% CI. There were no missing data, and *P* < .05 was considered significant. Analyses were performed in Python version 3.12 (Python Software Foundation) using the statsmodels, SciPy, and scikit-posthocs packages. A bar chart (Fig. 1) shows the percentage of providers reporting each challenge and resource need.

**Figure 1.**
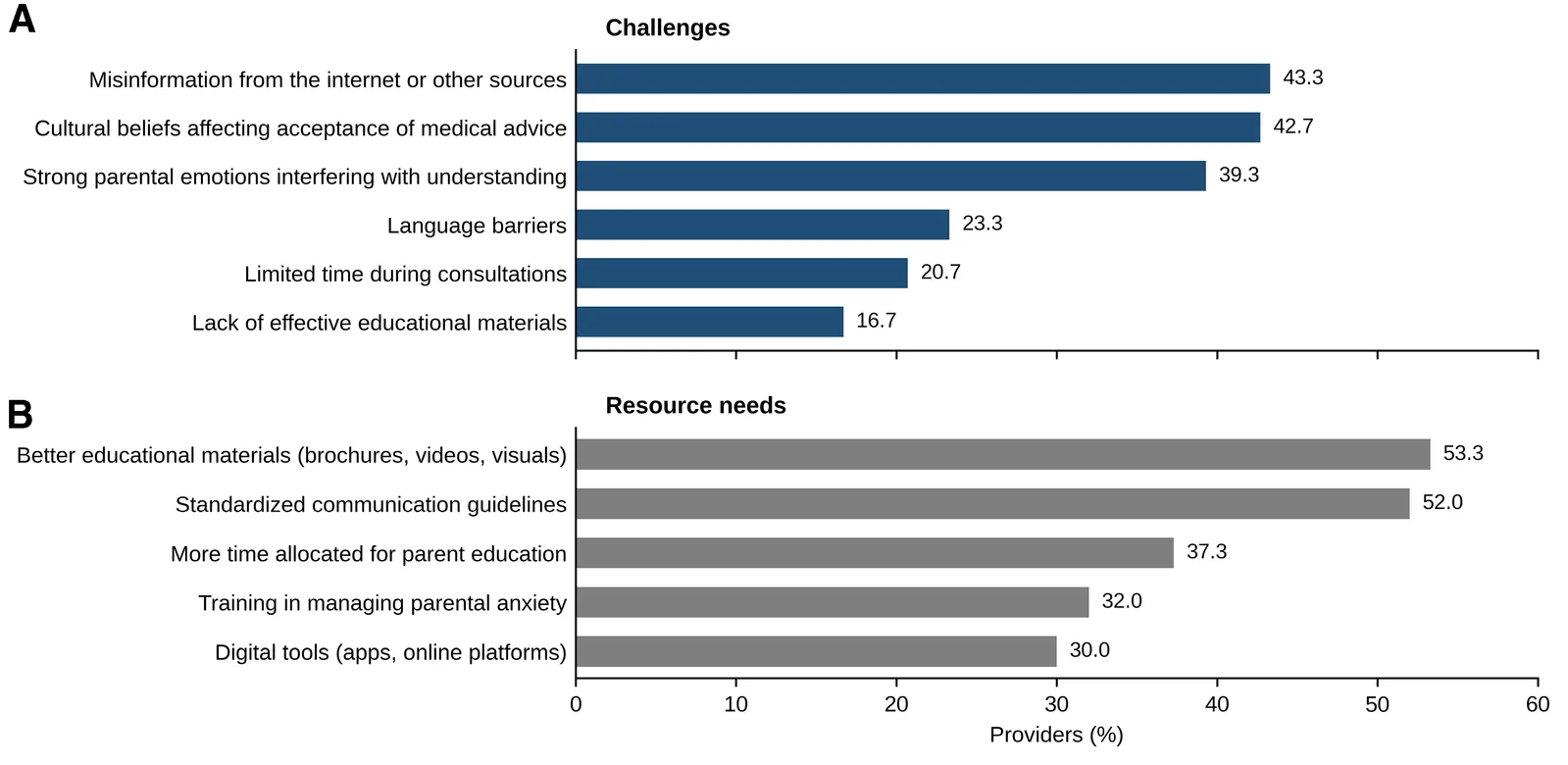
Percentage of providers reporting each challenge (A) and resource need (B) (n = 150). More than 1 answer was allowed.

### 2.5. Ethical consideration

The study received ethical approval from the University of Tabuk Institutional Ethics Committee (approval no. UT-583-360-2025). Participants received information about the study before answering. Participation was entirely voluntary, anonymous, and completion of the questionnaire was considered informed consent. All collected data remained confidential and were used exclusively for research purposes.

## 3. Results

### 3.1. Demographics, communication practices, and training background

The study involved 150 healthcare providers. Healthcare providers represented a variety of clinical practices, with pediatricians being the largest subgroup (54.0%, 95% CI: 46.0–61.8) and emergency physicians the least represented (6.0%, 95% CI: 3.2–11.0). A clinical practice of >10 years was the most common, whereas <1 year of experience was uncommon. General hospitals were the primary workplace setting, compared to private clinic/hospital, which accounted for the smallest proportion. The most frequently reported range of FS cases per month was 5 to 10.

Verbal explanation and guideline use were the most common communication practices. Most providers (95, 63.3%) rated their confidence 4 or 5 on the 5-point scale (median 4, interquartile range 3–5). Communication guidelines were used by 128 providers (85.3%, 95% CI: 78.8–90.1). Formal communication training varied: 79 providers (52.7%) had received training during their studies or through workshops or continuing medical education, 49 (32.7%) relied on experience, and 22 (14.7%) had no training at all. About half of providers (77, 51.3%) spent 5 to 10 minutes on counseling. Verbal explanation was the main teaching method (138, 92.0%; 95% CI: 86.5–95.4), whereas demonstrations (29, 19.3%) and visual aids, videos, digital tools, printed brochures, and support groups (each 12.0% or less) were used infrequently.

The points covered most often were the causes and risk factors of FS and the steps to take during a seizure (115 each, 76.7%), followed by the definition of FS (114, 76.0%). When to seek emergency care was covered least (70, 46.7%), and 37 providers (24.7%) covered all 7 points. To check parents’ understanding, about half of providers asked them to repeat key points (76, 50.7%), 46 (30.7%) observed their reactions, and 16 (10.7%) used no formal method. Fear was the feeling providers encountered most often (74, 49.3%), followed by satisfaction after clarification (46, 30.7%). Detailed demographic characteristics, communication practices, and training background are shown in Table 1.

**Table 1:** Characteristics, communication practices, and counseling content reported by healthcare providers (n = 150).

Variable	Category	n (%)	95% CI (%)
A. Demographic and professional characteristics			
Profession	Pediatrician	81 (54.0)	46.0–61.8
	Nurse	46 (30.7)	23.8–38.5
	General practitioner	14 (9.3)	5.6–15.1
	Emergency physician	9 (6.0)	3.2–11.0
Years of experience	<1 yr	14 (9.3)	5.6–15.1
	1–5 yr	43 (28.7)	22.0–36.4
	6–10 yr	29 (19.3)	13.8–26.4
	>10 yr	64 (42.7)	35.0–50.7
Primary workplace	General hospital	73 (48.7)	40.8–56.6
	Pediatric hospital	58 (38.7)	31.2–46.7
	Private clinic/hospital	19 (12.7)	8.3–18.9
Febrile seizure cases per month	<5 cases	58 (38.7)	31.2–46.7
	5–10 cases	62 (41.3)	33.8–49.3
	11–20 cases	10 (6.7)	3.7–11.8
	>20 cases	20 (13.3)	8.8–19.7
B. Communication practices and training			
Confidence in educating parents	Score 1 (not confident)	6 (4.0)	1.8–8.5
	Score 2	21 (14.0)	9.3–20.5
	Score 3	28 (18.7)	13.2–25.7
	Score 4	39 (26.0)	19.6–33.6
	Score 5 (very confident)	56 (37.3)	30.0–45.3
Use of guidelines	Yes	128 (85.3)	78.8–90.1
	No	22 (14.7)	9.9–21.2
Formal training received	Workshops or CME	34 (22.7)	16.7–30.0
	Training during medical/nursing school	45 (30.0)	23.2–37.8
	No formal training (experience-based)	49 (32.7)	25.7–40.5
	No training at all	22 (14.7)	9.9–21.2
Time spent educating parents	<5 min	21 (14.0)	9.3–20.5
	5–10 min	77 (51.3)	43.4–59.2
	10–15 min	39 (26.0)	19.6–33.6
	>15 min	13 (8.7)	5.1–14.3
Methods used to educate parents (more than 1 allowed)	Verbal explanation	138 (92.0)	86.5–95.4
	Demonstrations (e.g., recovery position)	29 (19.3)	13.8–26.4
	Visual aids (drawings and diagrams)	18 (12.0)	7.7–18.2
	Support groups/helplines	18 (12.0)	7.7–18.2
	Videos or animations	17 (11.3)	7.2–17.4
	Digital tools (apps/websites)	15 (10.0)	6.2–15.8
	Printed brochures	14 (9.3)	5.6–15.1
C. Counseling content, checking understanding, and feelings encountered			
Key points covered (>1 allowed)	Causes and risk factors	115 (76.7)	69.3–82.7
	Steps to take during a seizure	115 (76.7)	69.3–82.7
	Definition of febrile seizures	114 (76.0)	68.6–82.1
	How to recognize febrile seizures	101 (67.3)	59.5–74.3
	Long-term risks and likelihood of recurrence	82 (54.7)	46.7–62.4
	Misconceptions about febrile seizures	73 (48.7)	40.8–56.6
	When to seek emergency care	70 (46.7)	38.9–54.6
	All 7 points covered	37 (24.7)	18.5–32.1
Checking parental understanding	Asking them to repeat key points	76 (50.7)	42.7–58.6
	Observing their reactions and facial expressions	46 (30.7)	23.8–38.5
	No formal evaluation method	16 (10.7)	6.7–16.6
	Scheduling follow-up appointments	7 (4.7)	2.3–9.3
	Providing a quiz or checklist	5 (3.3)	1.4–7.6
Feeling most often encountered	Fear	74 (49.3)	41.4–57.3
	Satisfaction after clarification	46 (30.7)	23.8–38.5
	Confusion	24 (16.0)	11.0–22.7
	Anger/frustration	4 (2.7)	1.0–6.7
	Guilt	1 (0.7)	0.1–3.7
	Lack of interest	1 (0.7)	0.1–3.7

Percentages are of all 150 providers; for items allowing more than 1 answer, percentages add to >100%.

CI = confidence interval (Wilson method), CME = continuing medical education.

### 3.2. Challenges and resource needs

Table 2 and Figure 1 present the challenges healthcare providers face when communicating with parents about FS, along with the resources they believe would enhance this process. Misinformation (65, 43.3%), cultural beliefs affecting acceptance of medical advice (64, 42.7%), and strong parental emotions (59, 39.3%) were the challenges providers mentioned most often, and lack of effective educational materials was mentioned least (25, 16.7%). More than half of providers (81, 54.0%) reported 2 or more challenges. Providers most often asked for better educational materials (80, 53.3%) and standardized communication guidelines (78, 52.0%), followed by more time allocated for parent education (56, 37.3%), training in managing parental anxiety (48, 32.0%), and digital tools (45, 30.0%).

**Table 2:** Challenges and resource needs reported by healthcare providers (>1 answer allowed).

Item	n (%)	95% CI (%)
A. Challenges		
Misinformation from the internet or other sources	65 (43.3)	35.7–51.3
Cultural beliefs affecting acceptance of medical advice	64 (42.7)	35.0–50.7
Strong parental emotions interfering with understanding	59 (39.3)	31.9–47.3
Language barriers	35 (23.3)	17.3–30.7
Limited time during consultations	31 (20.7)	15.0–27.8
Lack of effective educational materials	25 (16.7)	11.6–23.4
Two or more challenges reported	81 (54.0)	46.0–61.8
B. Resource needs		
Better educational materials (brochures, videos, and visuals)	80 (53.3)	45.4–61.1
Standardized communication guidelines	78 (52.0)	44.1–59.8
More time allocated for parent education	56 (37.3)	30.0–45.3
Training in managing parental anxiety	48 (32.0)	25.1–39.8
Digital tools (apps and online platforms)	45 (30.0)	23.2–37.8
Two or more resources requested	80 (53.3)	45.4–61.1

Percentages are of all 150 providers and add to >100%.

CI = confidence interval (Wilson method).

### 3.3. Communication outcomes across provider characteristics

Table 3 shows the unadjusted associations between provider characteristics and the 4 communication outcomes, and Table 4 gives the outcomes by profession and by workplace. Pediatricians reported higher confidence than other professions (*P* = .004), with the difference lying between pediatricians and nurses (Bonferroni-adjusted *P* = .005). Profession was not associated with guideline use, formal training, or counseling time. Workplace was associated with formal training (*P* = .004), confidence (*P* = .027), and counseling time (*P* = .031). Providers in pediatric hospitals rarely reported having no communication training (1.7% vs 24.7% in general hospitals; adjusted *P* = .005), and providers in private clinics/hospitals most often counseled for 10 minutes or longer (57.9% vs 24.7% in general hospitals; adjusted *P* = .029). No pairwise workplace difference in confidence remained after Bonferroni correction. Guideline use differed by experience (*P* = .011), from 64.3% among providers with <1 year to 95.3% among those with >10 years. Confidence also differed by experience (*P* < .001) and by febrile seizure cases per month (*P* = .010), and guideline use by febrile seizure cases per month (*P* = .023).

**Table 3:** Unadjusted associations between provider characteristics and communication outcomes (*P* values).

Provider characteristic	Guideline use	Formal training	Confidence	Counseling time
Profession	.618	.206	.004	.818
Years of experience	.011	.345	<.001	.332
Primary workplace	.067	.004	.027	.031
Febrile seizure cases per month	.023	.424	.010	.378

Monte Carlo exact chi-square test (20,000 permutations) for guideline use and formal training (4 categories); Kruskal–Wallis test for confidence (5-point score) and counseling time (4 ordered categories). Significance: *P* < .05. Group values and post hoc results are given in Table 4 and the text.

**Table 4:** Communication outcomes by profession and by workplace.

Group	n	Confidence, median (IQR)	Confidence 4 or 5, n (%)	Counseling 10 min or more, n (%)	Any formal training, n (%)	Uses guidelines, n (%)
Profession						
Pediatrician	81	5 (3–5)	59 (72.8)	29 (35.8)	44 (54.3)	69 (85.2)
Nurse	46	3.5 (3–4)	23 (50.0)	15 (32.6)	24 (52.2)	39 (84.8)
General practitioner	14	4 (3–4.75)	9 (64.3)	6 (42.9)	8 (57.1)	11 (78.6)
Emergency physician	9	3 (2–4)	4 (44.4)	2 (22.2)	3 (33.3)	9 (100)
*P* (profession)		.004		.818	.206	.618
Primary workplace						
General hospital	73	4 (3–5)	43 (58.9)	18 (24.7)	31 (42.5)	63 (86.3)
Pediatric hospital	58	4 (3.25–5)	43 (74.1)	23 (39.7)	37 (63.8)	52 (89.7)
Private clinic/hospital	19	3 (2–5)	9 (47.4)	11 (57.9)	11 (57.9)	13 (68.4)
*P* (workplace)		.027		.031	.004	.067

Confidence was rated from 1 (not confident) to 5 (very confident). Any formal training = training during professional studies or through workshops/continuing medical education. *P* values: Kruskal–Wallis test for confidence and counseling time (4 ordered categories); Monte Carlo exact chi-square test (20,000 permutations) for formal training (4 categories) and guideline use. Post hoc results are given in the text.

IQR = interquartile range.

### 3.4. Adjusted associations with communication outcomes

Table 5 shows the adjusted associations between provider characteristics and the 4 communication outcomes. Guideline use was associated with >10 years of experience (aOR 6.97, 95% CI: 2.08–30.08) and with seeing 5 or more febrile seizure cases per month (aOR 4.04, 95% CI: 1.54–11.46). Providers in pediatric hospitals were more likely to have had formal communication training (aOR 2.44, 95% CI: 1.17–5.08). Pediatricians (aOR 2.40, 95% CI: 1.27–4.54) and providers with >10 years of experience (aOR 4.20, 95% CI: 2.08–8.52) reported higher confidence, whereas workplace was not independently associated with confidence (pediatric hospital aOR 1.87, 95% CI: 0.97–3.62). Providers in private clinics/hospitals counseled longer than those in general hospitals (aOR 3.81, 95% CI: 1.35–10.79). Sensitivity analyses gave the same significant associations, with 2 exceptions: in the binary confidence model, the pediatrician estimate was similar but not significant (aOR 2.03, *P* = .062), and in the 5-level confidence model, seeing >5 cases/mo also reached significance (aOR 1.93, *P* = .040).

**Table 5:** Multivariable models of communication outcomes, adjusted odds ratio (95% CI).

Predictor (reference)	Guideline use (Firth)	Any formal training	Higher confidence (ordinal)	Longer counseling time (ordinal)
Pediatrician (other professions)	0.74 (0.26–2.02)	0.98 (0.49–1.96)	2.40 (1.27–4.54)*	1.16 (0.61–2.23)
Experience 6–10 yr (≤5 yr)	1.63 (0.53–5.68)	0.97 (0.38–2.45)	2.20 (0.96–5.06)	1.45 (0.62–3.40)
Experience >10 yr (≤5 yr)	6.97 (2.08–30.08)*	1.09 (0.51–2.31)	4.20 (2.08–8.52)*	1.16 (0.58–2.34)
Pediatric hospital (general hospital)	1.47 (0.48–4.81)	2.44 (1.17–5.08)*	1.87 (0.97–3.62)	1.50 (0.76–2.94)
Private clinic/hospital (general hospital)	0.36 (0.10–1.31)	1.90 (0.67–5.37)	0.60 (0.22–1.67)	3.81 (1.35–10.79)*
≥5 FS cases/mo (<5)	4.04 (1.54–11.46)*	1.54 (0.78–3.04)	1.81 (0.97–3.39)	1.81 (0.96–3.43)
Model check	Nagelkerke *R*^2^ 0.25; Hosmer–Lemeshow *P* = .99	Nagelkerke *R*^2^ 0.07; Hosmer–Lemeshow *P* = .52	Brant test *P* = .070	Brant test *P* = .124

All 4 provider characteristics were entered in each model (n = 150). For the ordinal models, an aOR above 1 means higher odds of a higher confidence score or a longer counseling time. Variance inflation factors ranged from 1.02 to 1.28. Nagelkerke *R*^2^ and the Hosmer–Lemeshow test for guideline use are from the standard logistic model, whose estimates were similar to those of the Firth model.

aOR = adjusted odds ratio, CI = confidence interval, FS = febrile seizure.

* *P* < .05.

## 4. Discussion

This study examined the communication practices healthcare providers use when counseling parents about FS, and the challenges and resource needs they reported. Overall, guideline use was associated with longer experience and more frequent FS exposure. Confidence was higher among pediatricians and more experienced providers, formal training was more common in pediatric hospitals, and counseling lasted longer in private clinics/hospitals. Misinformation, cultural beliefs, and strong parental emotions were each cited by about 40% of providers, and better educational materials and standardized guidelines were the resources they requested most. Parental fear, often disproportionate to medical risk, was a recurring challenge, consistent with prior reports describing FS as one of the most anxiety-provoking events for caregivers.^[14,15]^ Emotional overload may impair parental comprehension during counseling, underscoring the need for communication approaches that validate emotions while delivering clear, structured information.

Misinformation was the challenge providers cited most often, consistent with earlier studies showing persistent misconceptions about FS and frequent reliance on unreliable online sources.^[16,17]^ Cultural beliefs were cited almost as often as misinformation, which suggests that correcting facts alone may not be enough and that educational materials also need to fit local beliefs and language. Although few providers named lack of educational materials as a challenge (16.7%), better materials were the resource they requested most (53.3%), and printed, visual, and digital materials were rarely used. The limited use of visual aids, videos, or digital resources in this study mirrors global findings of underutilized modern tools in pediatric education, despite evidence that audiovisual materials improve understanding and long-term retention.^[18,19]^

Formal communication training varied widely, and many providers relied on experience rather than structured training, consistent with gaps in communication curricula worldwide.^[19]^ Training was more common in pediatric hospitals but did not differ by profession. Pediatricians were more confident than nurses, yet they did not report more guideline use, training, or counseling time. Their confidence may therefore come mainly from clinical familiarity with FS rather than from communication training. Confidence was also higher among more experienced providers, and guideline use among those with more experience and more FS cases per month. Scenario-based simulations and supervised counseling practice may help less experienced staff, and those who see FS less often, build these skills.^[20]^ Providers in private clinics/hospitals counseled longest, possibly because they see fewer patients per clinic session, although our survey did not measure patient volume.

These findings carry several practical implications. Healthcare institutions should consider developing unified, culturally tailored educational materials addressing FS, as well as standardized counseling protocols to ensure consistent messaging. Integrating digital tools such as QR codes linked to videos or mobile applications may mitigate time constraints and reduce misinformation. Training modules focused on managing parental anxiety during acute events could further enhance counseling effectiveness. Whether these measures improve parental understanding or reduce parental anxiety was not assessed here and should be tested in studies that measure parent outcomes directly.

The strengths of this study are that it included 4 professional groups from 3 types of practice settings, assessed communication practices together with challenges and resource needs, and adjusted for confounding in multivariable models.

The study also has limitations. Its cross-sectional design cannot establish causal relationships between provider characteristics and communication practices. All data were self-reported, which introduces risks of recall and social desirability bias. Parental anxiety and understanding were not measured; our data describe what providers report doing, not how parents received the counseling. Convenience sampling at 5 facilities in a single city may limit how far the results apply to other settings, particularly rural or resource-limited areas. Online data collection may also have favored providers who are more comfortable with digital tools, and because the response rate could not be calculated, we could not assess nonresponse bias. The questionnaire was developed for this study and has not been validated against an external standard. Confidence was measured with a single self-rated item, which cannot capture every aspect of communication competence. Some subgroups were small (9 emergency physicians and 19 providers in private clinics/hospitals), which limits the precision of their estimates. Future studies should use validated multi-item tools and objective performance assessments, and should evaluate the effect of structured communication training, standardized materials, and digital interventions on parental understanding and anxiety. National multicenter research would show whether these findings apply more widely and would support unified communication guidelines for FS counseling.

## 5. Conclusion

In this survey of 150 providers in Tabuk, FS counseling relied mainly on verbal explanation, and printed, visual, and digital materials were rarely used. Experienced providers more often used communication guidelines and felt more confident; pediatricians were more confident than other professions, and formal communication training was more common in pediatric hospitals. Misinformation, cultural beliefs, and strong parental emotions were the barriers providers cited most often, and they asked mainly for standardized guidelines and better educational materials. Training and materials could be directed at less experienced staff and at general hospitals, where formal training was least common. Whether they improve parents’ understanding or reduce their anxiety should be tested in future studies.

## Acknowledgments

We would like to thank Editage (www.editage.com) for English language editing and journal submission support. The authors have authorized the submission of this manuscript through Editage.

## Author contributions

**Conceptualization:** Laila A. Albishi.

**Formal analysis:** Laila A. Albishi, Meshari Abdulrahman Y. Alharthi, Bdour Awad Almasoudi.

**Methodology:** Laila A. Albishi, Aseel Hameed Algohani, Reem Fayez J Alanazi, Layan Mohammed M. Alamri, Ftoon Sattam A. Albalawi, Raghad Ibrahim H. AlOmari, Ammar Mohammed Aeid, Rana Nafea Alanazi, Meshari Abdulrahman Y. Alharthi, Bdour Awad Almasoudi.

**Project administration:** Laila A. Albishi, Aseel Hameed Algohani, Reem Fayez J. Alanazi.

**Resources:** Laila A. Albishi, Raghad Ibrahim H. AlOmari, Ammar Mohammed Aeid, Rana Nafea Alanazi.

**Software:** Laila A. Albishi, Layan Mohammed M. Alamri, Ftoon Sattam A. Albalawi.

**Supervision:** Laila A. Albishi, Aseel Hameed Algohani.

**Validation:** Laila A. Albishi.

**Visualization:** Laila A. Albishi.

**Writing – original draft:** Laila A. Albishi.

**Writing – review & editing:** Laila A. Albishi, Aseel Hameed Algohani, Reem Fayez J. Alanazi, Layan Mohammed M. Alamri, Ftoon Sattam A. Albalawi, Raghad Ibrahim H. AlOmari, Ammar Mohammed Aeid, Rana Nafea Alanazi, Meshari Abdulrahman Y. Alharthi, Bdour Awad Almasoudi.


