# Supplementary material for: Communication practices and challenges of healthcare providers in febrile seizure counseling: A cross-sectional study
Source: Medicine (Baltimore). 2026 Oct 23;105(43):e51284. doi: 10.1097/MD.0000000000051284 (PMC13706436; doi:10.1097/MD.0000000000051284)
Supplement: Supplementary file 1 [file medi-105-e51284-s001.docx]

# Supplemental Digital Content 1

Research title:

Communication Practices and Challenges of Healthcare Providers in Febrile Seizure Counseling: A Cross-Sectional Study

# Study questionnaire

**Invitation**

**Dear Healthcare Provider**,

You are invited to participate in a research study examining how healthcare providers communicate with and counsel parents or caregivers of children experiencing febrile seizures. The study aims to understand current counseling practices, the information commonly provided to families, and the challenges healthcare providers may encounter when discussing febrile seizures with parents or caregivers. The study was approved by the University of Tabuk Institutional Ethics Committee (approval no. UT-583-360-2025).

Your participation will involve completing a brief online questionnaire. The questionnaire asks about your professional background, your usual communication and counseling practices, the information you provide to families, and any challenges or barriers you may experience during febrile seizure counseling.

Participation is **voluntary**. You may choose not to participate or stop completing the questionnaire at any time, without any consequences. You do not need to provide your name or other directly identifying information. All responses will be treated confidentially and will be used for **research purposes only**. The findings will be reported in aggregate form, so individual participants will not be identified.

There are no expected direct risks from participating in this study. Your responses may help provide a better understanding of current healthcare-provider practices and communication needs related to febrile seizure counseling.

The questionnaire takes approximately **5–10 minutes** to complete.

By clicking “Agree,” you confirm your consent to participate in this research study.

**Do you agree to participate in this research study?**

☐ Agree

☐ Disagree

**Thank you for your time and contribution to this research.**

**Primary investigator**	Dr. Laila A. AlBishi	Faculty of Medicine, University of Tabuk

## Section 1: Demographic data

1. What is your profession?
   - ○ Pediatrician
   - ○ Nurse
   - ○ General practitioner
   - ○ Emergency physician
2. How many years of clinical experience do you have?
   - ○ <1 year
   - ○ 1–5 years
   - ○ 6–10 years
   - ○ More than 10 years
3. What is your primary workplace?
   - ○ General hospital
   - ○ Pediatric hospital
   - ○ Private clinic/hospital
4. How many febrile seizure cases do you see per month?
   - ○ <5 cases
   - ○ 5–10 cases
   - ○ 11–20 cases
   - ○ More than 20 cases

## Section 2: Knowledge and confidence in febrile seizure communication

5. How confident are you in educating parents about febrile seizures? (1 = not confident, 5 = very confident)
   - ○ 1
   - ○ 2
   - ○ 3
   - ○ 4
   - ○ 5
6. Do you use specific communication guidelines when counseling parents about febrile seizures?
   - ○ Yes
   - ○ No
7. Which key points do you cover when explaining febrile seizures to parents? (select all that apply)
   - ☐ Definition of febrile seizures
   - ☐ Causes and risk factors
   - ☐ How to recognize febrile seizures
   - ☐ Steps to take during a seizure
   - ☐ When to seek emergency care
   - ☐ Long-term risks and likelihood of recurrence
   - ☐ Misconceptions about febrile seizures
8. Have you received formal training in communicating with parents about febrile seizures?
   - ○ Training during medical/nursing school
   - ○ Workshops or continuing medical education (CME)
   - ○ No formal training (experience-based)
   - ○ No training at all

## Section 3: Communication strategies

9. Which methods do you use to educate parents about febrile seizures? (select all that apply)
   - ☐ Verbal explanation
   - ☐ Demonstrations (e.g., recovery position)
   - ☐ Visual aids (drawings, diagrams)
   - ☐ Videos or animations
   - ☐ Digital tools (apps/websites)
   - ☐ Printed brochures
   - ☐ Support groups/helplines
10. How much time do you usually spend educating parents about febrile seizures during a consultation?
    - ○ <5 minutes
    - ○ 5–10 minutes
    - ○ 10–15 minutes
    - ○ >15 minutes
11. How do you check whether parents have understood your explanation?
    - ○ Asking them to repeat key points
    - ○ Observing their reactions and facial expressions
    - ○ Providing a quiz or checklist
    - ○ Scheduling follow-up appointments
    - ○ No formal evaluation method
12. Which feeling do you most often encounter when discussing febrile seizures with parents?
    - ○ Fear
    - ○ Confusion
    - ○ Guilt
    - ○ Anger/frustration
    - ○ Satisfaction after clarification
    - ○ Lack of interest

## Section 4: Challenges in communicating with parents

13. What are the main challenges you face when educating parents about febrile seizures? (select all that apply)
    - ☐ Language barriers
    - ☐ Strong parental emotions interfering with understanding
    - ☐ Misinformation from the internet or other sources
    - ☐ Limited time during consultations
    - ☐ Cultural beliefs affecting acceptance of medical advice
    - ☐ Lack of effective educational materials
14. Which additional resources would help you improve communication with parents? (select all that apply)
    - ☐ Better educational materials (brochures, videos, visuals)
    - ☐ Standardized communication guidelines
    - ☐ More time allocated for parent education
    - ☐ Training in managing parental anxiety
    - ☐ Digital tools (apps, online platforms)

## Response format and coding in the analysis

| Item | Response format | Coding in the analysis |
| --- | --- | --- |
| 1. Profession | Single choice | 4 categories; pediatrician vs other in regression models |
| 2. Clinical experience | Single choice | 4 categories; 5 years or less, 6-10, more than 10 in regression models |
| 3. Primary workplace | Single choice | 3 categories (general hospital as reference in regression models) |
| 4. Febrile seizures per month | Single choice | 4 categories; fewer than 5 vs 5 or more in regression models |
| 5. Confidence | 5-point rating scale | Outcome; ordinal score 1 to 5 (1 and 2 combined in the ordinal model) |
| 6. Guideline use | Yes/no | Outcome; binary |
| 7. Key points covered | Select all that apply | Collected; not analyzed in this report |
| 8. Formal training | Single choice | Outcome; 4 categories, and any formal training (studies or workshops/CME) vs none (experience only or no training) |
| 9. Teaching methods | Select all that apply | Each option coded 0/1; percentage of all 150 respondents (Table 1) |
| 10. Counseling time | Single choice | Outcome; 4 ordered categories |
| 11. Checking parental understanding | Single choice | Collected; not analyzed in this report |
| 12. Feelings during discussion | Single choice | Collected; not analyzed in this report |
| 13. Challenges | Select all that apply | Each option coded 0/1; percentage of all 150 respondents (Table 2) |
| 14. Resource needs | Select all that apply | Each option coded 0/1; percentage of all 150 respondents (Table 2) |

CME, continuing medical education.
